# Low-dose aspirin confers a survival benefit in patients with pathological advanced-stage oral squamous cell carcinoma

**DOI:** 10.1038/s41598-021-96614-y

**Published:** 2021-08-25

**Authors:** Sheng-Dean Luo, Shao-Chun Wu, Wei-Chih Chen, Ching-Nung Wu, Tai-Jan Chiu, Yao-Hsu Yang, Shau-Hsuan Li, Fu-Min Fang, Tai-Lin Huang, Chang-Chun Hsiao, Chang-Han Chen

**Affiliations:** 1 Department of Otolaryngology, Kaohsiung Chang Gung Memorial Hospital and Chang Gung University College of Medicine, Kaohsiung, 833 Taiwan; 2 Graduate Institute of Clinical Medical Sciences, College of Medicine, Chang Gung University, No. 259, Wenhua 1st Rd., Guishan District, Taoyüan, 333 Taiwan; 3 Department of Anesthesiology, Kaohsiung Chang Gung Memorial Hospital and Chang Gung University College of Medicine, Kaohsiung, 833 Taiwan; 4 Department of Hematology-Oncology, Kaohsiung Chang Gung Memorial Hospital and Chang Gung University College of Medicine, Kaohsiung, 833 Taiwan; 5 Department of Traditional Chinese Medicine, Chang Gung Memorial Hospital, Chiayi, Taiwan; 6 Health Information and Epidemiology Laboratory of Chang Gung Memorial Hospital, Chiayi, Taiwan; 7 School of Traditional Chinese Medicine, College of Medicine, Chang Gung University, Taoyüan, Taiwan; 8 Department of Radiation Oncology, Kaohsiung Chang Gung Memorial Hospital and Chang Gung University College of Medicine, Kaohsiung, Taiwan; 9 Division of Pulmonary and Critical Care Medicine, Kaohsiung Chang Gung Memorial Hospital and Chang Gung University College of Medicine, Kaohsiung, 83301 Taiwan; 10 Department of Applied Chemistry, and Graduate Institute of Biomedicine and Biomedical Technology, National Chi Nan University, Nantou, 54561 Taiwan; 11 Institute of Medicine, Chung Shan Medical University, Taichung, Taiwan; 12 Department of Medical Research, Chung Shan Medical University Hospital, Taichung City, 40201 Taiwan

**Keywords:** Cancer, Oral cancer

## Abstract

Oral squamous cell carcinoma (OSCC) remains one of the most challenging clinical problems in the field due to its high rate of locoregional and distant metastases. However, studies that assess the association between aspirin use and survival in patients with OSCC are limited. Moreover, patients that recruited from those studies might have tumors that arose from different anatomic regions of the head and neck, including the oral cavity, oropharynx, etc. Since tumors within these distinct anatomic regions are unique in the context of epidemiology and tumor progression, we sought to evaluate the association of aspirin use with squamous cell carcinomas located within the oral cavity only. In this 10-year cohort study, we evaluated aspirin use and survival rates in relation to clinical characteristics as well as duration of aspirin use in patients with OSCC. Our findings suggest that OSCC patients with aspirin use for more than 180 days showed improved overall and disease-specific survival rates. Aspirin also improves survival in patients across various stages of OSCC. Cox regression models indicated that aspirin use was associated with a good prognosis. In conclusion, this evidence indicates that aspirin may be potentially used as an adjuvant therapy for OSCC.

## Introduction

Oral squamous cell carcinoma (OSCC) is a common malignant tumor in the oral cavity areas including the lip, tongue, palate, etc^1,2^. It accounts for approximately 80–90% of all cases of oral cancer^1,2^. According to a report of the World Health Organization (WHO) on oral cancer, the incidence rates of oral cancer are high, and it remains responsible for the top three most common cancers in certain Asian-Pacific countries^3,4^. Although its prevalence is low in Western countries, the incidence rates of oral cancer driven by human papillomavirus (HPV) infections and some lifestyle habits have increased gradually but significantly and become a public health concern^5–10^.

Adopting unhealthy lifestyle habits such as tobacco and alcohol consumption is the primary cause of oral cancer in Western countries^8–10^. In Asian-Pacific countries, on top of tobacco and alcohol consumption, betel (areca) nut chewing is also known as one of the major factors contributing to the risk of developing oral cancer^11,12^. Indeed, accumulating evidence has demonstrated that adopting these unhealthy lifestyle habits, as well as HPV infection and poor oral hygiene, have significant associations with chronic inflammation that can, in turn, act as a driving force in increasing the risk of oral cancer development^13–15^.

Aspirin is a well-known nonsteroidal anti-inflammatory drug (NSAID) that has been commonly used to reduce pain and inflammation from a spectrum of inflammatory disorders^14^. Given that the anti-inflammatory properties of aspirin are correlated with inhibition of tumor progression across multiple cancer types, it may exert similar effects on OSCC since overwhelming chronic inflammation has a pivotal role in the development of this cancer^13,14^. In particular, aspirin exhibits as an effective drug for blockage of COX-2 and PGE2. This makes it more plausible that aspirin use will benefit patients with OSCC due to a higher expression of COX-2 in patients with OSCC^14^. For these reasons, our interest in investigating whether aspirin can improve the clinical outcomes of OSCC patients was piqued. In addition, studies of the association between aspirin use and survival in patients with OSCC are lacking. To that end, we used the database from Chang Gung Memorial Hospital over a 10-year follow-up period involving 1,525 participants to analyze the correlation of aspirin use with survival rates in relation to clinical characteristics as well as duration of aspirin use in patients with OSCC.

## Methods

### Patient recruitment

This cohort study was reviewed and approved by the Institutional Review Board (IRB) of the Kaohsiung and Chiayi branches of Chang Gung Memorial Hospital. According to these protocols, informed consent was waived due to the nature of the study design and IRB regulations. All methods were performed in accordance with the relevant guidelines and regulations. All experimental protocols were approved by the Kaohsiung and Chiayi branches of Chang Gung Memorial Hospital. The IRB approval protocol numbers are 201801348B0 and 201700253B0C602. This cohort comprises a total of 7,763 patients who were diagnosed with oral cavity cancer in Chang-Gung Memorial Hospital, Taiwan between January 2007 and December 2017. Exclusion criteria were clinical and pathological AJCC stage IVc or missing data on pathological stages of cancer (pathological AJCC or TN staging system), aspirin use for less than 180 days, oral cavity cancer with morphology codes other than squamous cell carcinoma (ICD-9-CM 140, 141, 143–145), and individuals who did not receive surgery or any treatment. In total, 1525 patients were analyzed in our study. The criterion for recruited patients in this study who had habits such as smoking, betel nut chewing, and alcohol consumption and comorbidities such as atrial fibrillation, DM, hypertension, and hyperlipidemia are listed in Table S1.

### Aspirin initiation and follow-up

In our study, low-dose aspirin (100 mg/day) was prescribed to patients who initially presented with cardiovascular and/or metabolic comorbidities at the time of OSCC diagnosis or during the time of follow-up care. We were able to trace the electronic records for the duration of aspirin treatment of the OSCC patients within a 10-year follow-up period. A ≥ 180-day definition period was applied to aspirin users who initiated aspirin use when they were diagnosed with OSCC, while non-users were those who did not initiate aspirin use when they were diagnosed with OSCC. For example, if Patient A was prescribed aspirin due to having comorbidity during a follow-up appointment that was 100 days after diagnosis of OSCC, then a ≥ 180-day definition period was started counting from day 100, and this day was also defined as the first survival day of Patient A. For Patient B, who was diagnosed with OSCC on the same day as Patient A but did not have an aspirin prescription, then the survival day of Patient B on day 100 after diagnosis of OSCC was 100 days. Although the way that we defined the survival of aspirin users was more stringent compared to non-users, our results still suggested that there is a beneficial effect of aspirin on the survival of patients with OSCC. Continuation of aspirin use for less than 180 days among aspirin users during the follow-up period did not confer a survival benefit. For this reason, patients who took aspirin when they were diagnosed with OSCC but whose continuation of aspirin use was less than 180 days were excluded. For analyses comparing 180-day aspirin users and non-users in this paper, the entire groups of aspirin users (N = 305) and nonusers (N = 1220) were followed up after being diagnosed with OSCC. The follow-up was ended on death. The median follow-up time was 56.9 (27.4–86.0) months (Table S2).

### Statistical analyses

Categorical variables including gender, comorbidities, lifestyle risk factors, pathological AJCC stages of cancer, etc. were tested by either a two-sided Fisher’s exact test or a Pearson’s chi-squared test. The normally and non-normally distributed continuous data were analyzed using Student’s 2-tailed t-tests and Mann–Whitney U tests, respectively. In order to minimize the confounding effect of groups that are comparable due to non-randomized allocations, a Mahalanobis 1:4 propensity score-matched (PSM) study group was performed using the Greedy method with a 0.25 caliper-width. We used NCSS 10 software (NCSS Statistical Software, Kaysville, UT, USA) with the setting mentioned above to test if a balanced covariate distribution between aspirin users and non-users was achieved by using a logistic regression model with the following covariates: gender, age, pathological AJCC stages of cancer and treatment. We excluded one unmatched aspirin user. The standardized mean difference (SMD) was less than 0.1 after 1:4 PSM, suggesting a good balance of covariate distribution was achieved between aspirin users and non-users (Table S3). The Kaplan–Meier method was used to evaluate the effects of aspirin use in the primary outcomes (OS and DSS) after adjusting for potential confounding variables such as gender, age, and pathological AJCC stages of cancer. A univariate analysis and Cox proportional-hazards model were used to evaluate any covariates that could affect the survival of OSCC patients. All statistical analyses were performed using SPSS Statistics V22.0 software for Windows.

## Results

### Demographic and clinical characteristics of patients diagnosed with OSCC

**Figure 1:**
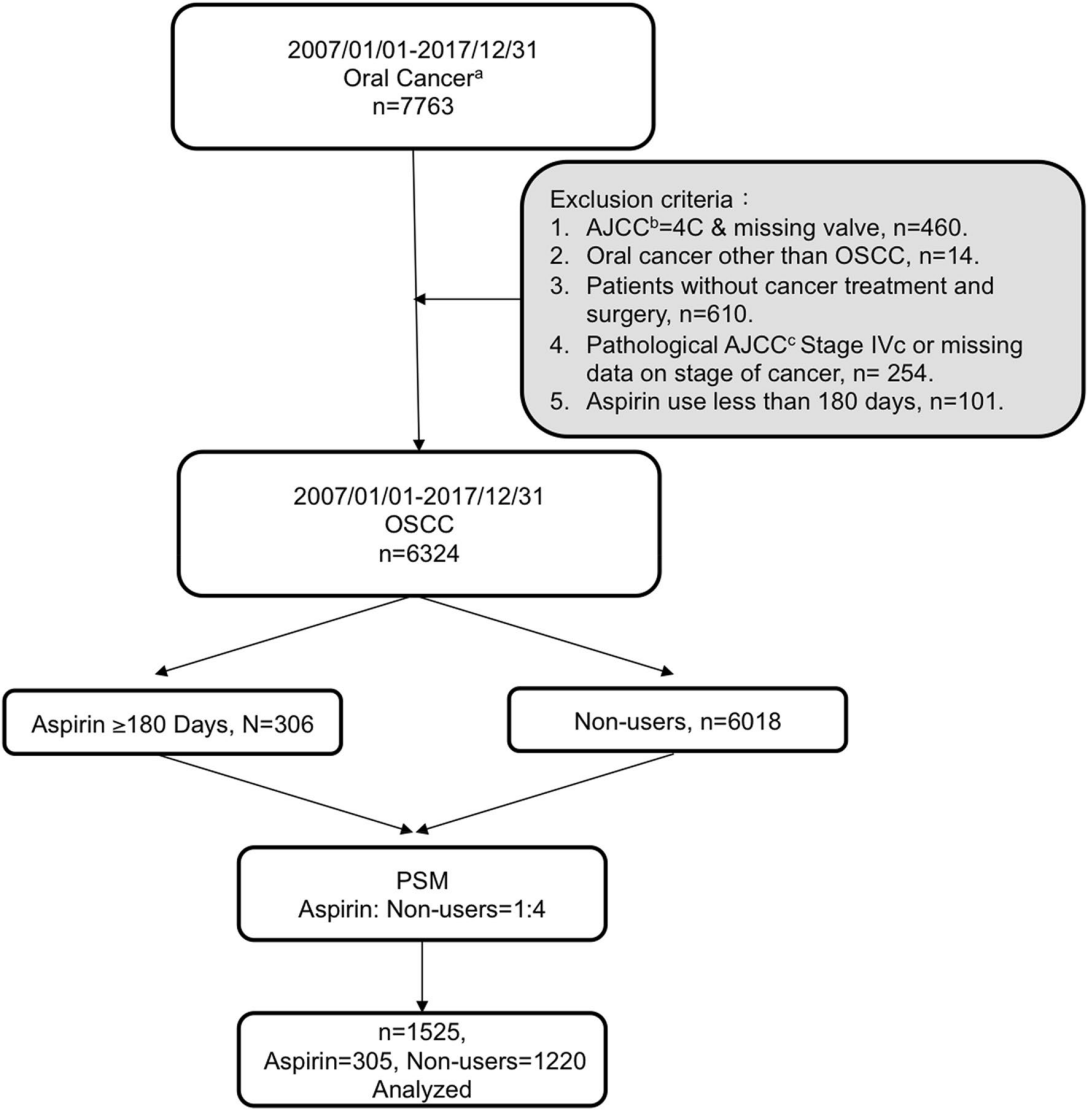
Flowchart showing the inclusion and exclusion criteria of the OSCC patients recruited to engage in this cohort study. A total of 7,763 patients who were diagnosed with oral cancer were recruited in this study. After matching aspirin users with non-users based on 1:4 propensity score analysis, there was a final inclusion of 1,525 OSCC patients were recruited for data analysis in this study. ^a^Clinical and Pathological AJCC Cancer Staging 7th Edition; ^b^Clinical AJCC Cancer Staging 7th Edition; ^c^Pathological AJCC Cancer Staging 7th Edition.

The flow chart in Fig. 1 demonstrates the strategy used to recruit OSCC patients for this cohort study that were admitted to Taiwan Chang Gung Memorial Hospital between 2007 and 2017. A total of 6324 OSCC patients who had neoplasms affecting regions within the oral cavity such as the tongue, buccal area, etc. were recruited after applying the exclusion criteria. OSCC patients with a history of aspirin use were propensity score matched (PSM) in a 1:4 ratio to those without aspirin use: 305 were aspirin users and 1220 were non-users after excluding one unmatched pair, i.e., 1 aspirin users and 4 non-users. The demographics and clinical characteristics of these 1525 patients (aspirin users and non-users) are presented in Table 1. The age, gender, pathological AJCC stages of cancer, cancer recurrence, treatment, and lifestyle risk factors of OSCC patients with aspirin use appeared similar to those of non-users. A statistically significant difference was seen between the aspirin users and non-users with regard to mortality, cause of death, and comorbidities (Table 1).

**Table 1:** Demographic and clinical characteristics of the cohort study (n = 1525).

Variables	N(%)/median (IQR)	Low-dose aspirin		*p* value
		Non-users	Users ≥ 180 days	
**Sex**				
Female	78(5.11%)	58(4.75%)	20(6.56%)	0.2010
Male	1447(94.89%)	1162(95.25%)	285(93.44%)	
**Age**				
Year	58(52–65)	58(52–65)	58(53–65)	0.7997
**Pathological stages of cancer (AJCC)**^**a**^				
I & II	892(58.49%)	711(58.28%)	181(59.34%)	0.7355
III & IV^b^	633(41.51%)	509(41.72%)	124(40.66%)	
**Cancer recurrence**				
No	1222(80.13%)	967(79.26%)	255(83.61%)	0.0890
Yes	303(19.87%)	253(20.74%)	50(16.39%)	
**Mortality**				
Alive	1059(69.44%)	816(66.89%)	243(79.67%)	< 0.0001
Death	466(30.56%)	404(33.11%)	62(20.33%)	
**Causes of death**				
Alive	1059(69.44%)	816(66.89%)	243(79.67%)	< 0.0001
Death due to OSCC	264(17.31%)	235(19.26%)	29(9.51%)	
Other-Causes of Death	202(13.25%)	169(13.85%)	33(10.82%)	
**Treatments**				
Surgery	857(56.2%)	681(55.82%)	176(57.7%)	0.5528
Surgery & RT or CCRT	668(43.8%)	539(44.18%)	129(42.3%)	
**Lifestyle risk factors**				
Smoking				
No	400(26.47%)	322(26.68%)	78(25.66%)	0.7187
Yes	1111(73.53%)	885(73.32%)	226(74.34%)	
Betel nut chewing				
No	637(42.16%)	504(41.76%)	133(43.75%)	0.5293
Yes	874(57.84%)	703(58.24%)	171(56.25%)	
Alcohol consumption				
No	665(44.01%)	529(43.83%)	136(44.74%)	0.7753
Yes	846(55.99%)	678(56.17%)	168(55.26%)	
**Comorbidities**				
Atrial fibrillation (flutter)				
No	1483(97.25%)	1203(98.61%)	280(91.8%)	< 0.0001
Yes	42(2.75%)	17(1.39%)	25(8.2%)	
DM				
No	1171(76.79%)	1023(83.85%)	148(48.52%)	< 0.0001
Yes	354(23.21%)	197(16.15%)	157(51.48%)	
Hypertension				
No	1082(70.95%)	987(80.9%)	95(31.15%)	< 0.0001
Yes	443(29.05%)	233(19.1%)	210(68.85%)	
Hyperlipidemia				
No	1221(80.07%)	1099(90.08%)	122(40.0%)	< 0.0001
Yes	304(19.93%)	121(9.92%)	183(60.0%)	

*IQR* Interquartile range, *RT* Radiotherapy, *CCRT* Concurrent chemoradiotherapy, *DM* Diabetes mellitus.

^a^Pathological AJCC Cancer Staging 7th Edition.

^b^Stages IVa and IVb only.

### Univariate and multivariate analyses of predictive variables for OSCC survival

The univariate Cox regression analysis indicated that pathological AJCC stages of cancer (HRstages I & II vs. stages III & IV = 4.22, 95% CI = 3.23–5.52, *p* < 0.0001), pathological T staging system (HRT1, 2 & 3 vs. T4 = 3.11, 95% CI = 2.43–3.98, *p* < 0.0001), pathological N staging system (HRN0 vs. N1, 2 & 3 = 4.25, 95% CI = 3.34–5.42, *p* < 0.0001), treatment (HRsurgery vs. surgery & RT or CCRT = 4.31, 95% CI = 3.27–5.68, *p* < 0.0001), aspirin use HRaspirin non-users vs. users = 0.44, 95% CI = 0.30–0.64, *p* < 0.0001), hyper-tension (HRno vs. yes = 0.68, 95% CI = 0.51–0.91, *p* = 0.0084), and hyperlipidemia (HRno vs. yes = 0.32, 95% CI = 0.21–0.48, *p* < 0.0001) were significant independent prognostic factors of survival. From the perspective of controlling for confounders, we further used three different multivariate Cox regression models (A, B, and C) to assess the influence of covariates on OSCC survival rates. In Model A (Table 2), which adjusted for covariates including age, gender, pathological AJCC stages of cancer, treatment, and aspirin use, the significant independent prognostic factors of survival were pathological AJCC stages of cancer (HRstages I & II vs. stages III & IV = 2.38, 95% CI = 1.69–3.35, *p* < 0.0001), treatment (HRsurgery vs. surgery & RT or CCRT = 2.55, 95% CI = 1.79–3.62, *p* < 0.0001), and aspirin use (HRaspirin non-users vs. users = 0.42, 95% CI = 0.29–0.63, *p* < 0.0001). In Model B (Table 3), which adjusted for covariates including age, gender, pathological TN stage of cancer, treatment and aspirin use, the significant independent prognostic factors of survival were pathological T staging system (HR T1, 2 & 3 vs. T4 = 1.57, 95% CI = 1.20–2.07, *p* = 0.0012), pathological N staging system (HRN0 vs. N1, 2 & 3 = 2.63, 95% CI = 1.99–3.47, *p* < 0.0001), treatment (HRsurgery vs. surgery & RT or CCRT = 2.30, 95% CI = 1.63–3.25, *p* < 0.0001), and aspirin use (HRaspirin non-users vs. users = 0.44, 95% CI = 0.30–0.64, *p* < 0.0001). In Model C (Table S4), which adjusted for covariates including age, gender, pathological AJCC stages of cancer, treatment, aspirin use and all comorbidities, the significant independent prognostic factors of survival were pathological AJCC stages of cancer (HRstages I & II vs. stages III & IV = 2.34, 95% CI = 1.66–3.30, *p* < 0.0001), treatment (HRsurgery vs. surgery & RT or CCRT = 2.60, 95% CI = 1.83–3.70, *p* < 0.0001), aspirin use (HRaspirin non-users vs. users = 0.59, 95% CI = 0.38–0.92, *p* = 0.0191, diabetes mellitus (HRno vs. yes = 1.65 (95% CI = 1.20–2.28, *p* = 0.0022) and hyperlipidemia (HRno vs. yes = 0.33, 95% CI = 0.20–0.53, *p* < 0.0001). Atrial fibrillation was not an independent prognostic factor for survival in either the univariate (HRno vs. yes = 0.80, 95% CI = 0.35–1.79, *p* = 0.5779) or multivariate Cox regression Model C (HRno vs. yes = 1.03, 95% CI = 0.45–2.40, *p* = 0.9376). Hypertension was no longer an independent prognostic factor for survival in multivariate Cox regression Model C (HRno vs. yes = 0.98, 95% CI = 0.71–1.34, *p* = 0.8841). All the univariate and multivariate Cox regression models used in the analyses indicated that age and gender were not independent prognostic factors for survival (Tables 2, 3, S2). Overall, all of our Cox regression models suggested that OSCC patients who used aspirin were associated with good prognoses, showing a decreased hazard of death that varied from 41 to 58% compared to non-users (Tables 2, 3, S2).

**Table 2:** Univariate and multivariate Cox proportional hazards of prognostic factors for OSCC survival, Model A: adjusted for pathological stages of cancer (AJCC) and other covariates as shown in the table.

Variables	Cohort n = 1525	Hazard ratio (95%CI)			
		Univariate	*p* value	Multivariate	*p* value
**Gender**					
Female	78(5.11%)	1	0.0843	1	0.3474
Male	1447(94.89%)	1.94(0.91–4.11)		1.44(0.67–3.07)	
**Age**					
Years	1525(100.00%)	1.00(0.99–1.01)	0.7096	1.01(1.00–1.02)	0.1167
**Pathological stages of cancer (AJCC)**^**a**^					
I & II	892(58.5%)	1	< 0.0001	1	< 0.0001
III & IV^b^	633(41.5%)	4.22(3.23–5.52)		2.38(1.69–3.35)	
**Treatments**					
Surgery	857(56.2%)	1	< 0.0001	1	< 0.0001
Surgery and RT or CCRT	668(43.8%)	4.31(3.27–5.68)		2.55(1.79–3.62)	
**Aspirin use**					
No	1220(80.0%)	1	< 0.0001	1	< 0.0001
Yes	305(20.0%)	0.44(0.30–0.64)		0.42(0.29–0.63)	

*95% CI* 95% Confidence interval, *RT* Radiotherapy, *CCRT* Concurrent chemoradiotherapy.

^a^Pathological AJCC Cancer Staging 7th Edition.

^b^Stages IVa and IVb only.

**Table 3:** Univariate and multivariate Cox proportional hazards of prognostic factors for OSCC survival, Model B: adjusted for pathological TN staging system and other covariates as shown in the table.

Variables	Cohort n = 1525	Hazard ratio (95%CI)			
		Univariate	*p* value	Multivariate	*p* value
**Gender**					
Female	78(5.11%)	1	0.0843	1	0.3384
Male	1447(94.89%)	1.94(0.91–4.11)		1.45(0.68–3.10)	
**Age**					
Years	1525(100.00%)	1.00(0.99–1.01)	0.7096	1.01(1.00–1.02)	0.0745
**Pathological T staging**^a^					
T1, 2 & 3	1219(79.93%)	1	< 0.0001	1	0.0012
T4	306(20.07%)	3.11(2.43–3.98)		1.57(1.20–2.07)	
**Pathological N staging**^b^					
N0	1140(74.75%)	1	< 0.0001	1	< 0.0001
N1, 2 & 3	385(25.25%)	4.25(3.34–5.42)		2.63(1.99–3.47)	
**Treatments**					
Surgery	857(56.2%)	1	< 0.0001	1	< 0.0001
Surgery and RT or CCRT	668(43.8%)	4.31(3.27–5.68)		2.30(1.63–3.25)	
**Aspirin use**					
No	1220(80.0%)	1	< 0.0001	1	< 0.0001
Yes	305(20.0%)	0.44(0.30–0.64)		0.44(0.30–0.64)	

*95% CI* 95% Confidence interval, *RT* Radiotherapy, *CCRT* Concurrent chemoradiotherapy.

^a^Pathological T Staging 7th Edition.

^b^Pathological N Staging 7th Edition.

### Survival analyses

**Figure 2:**
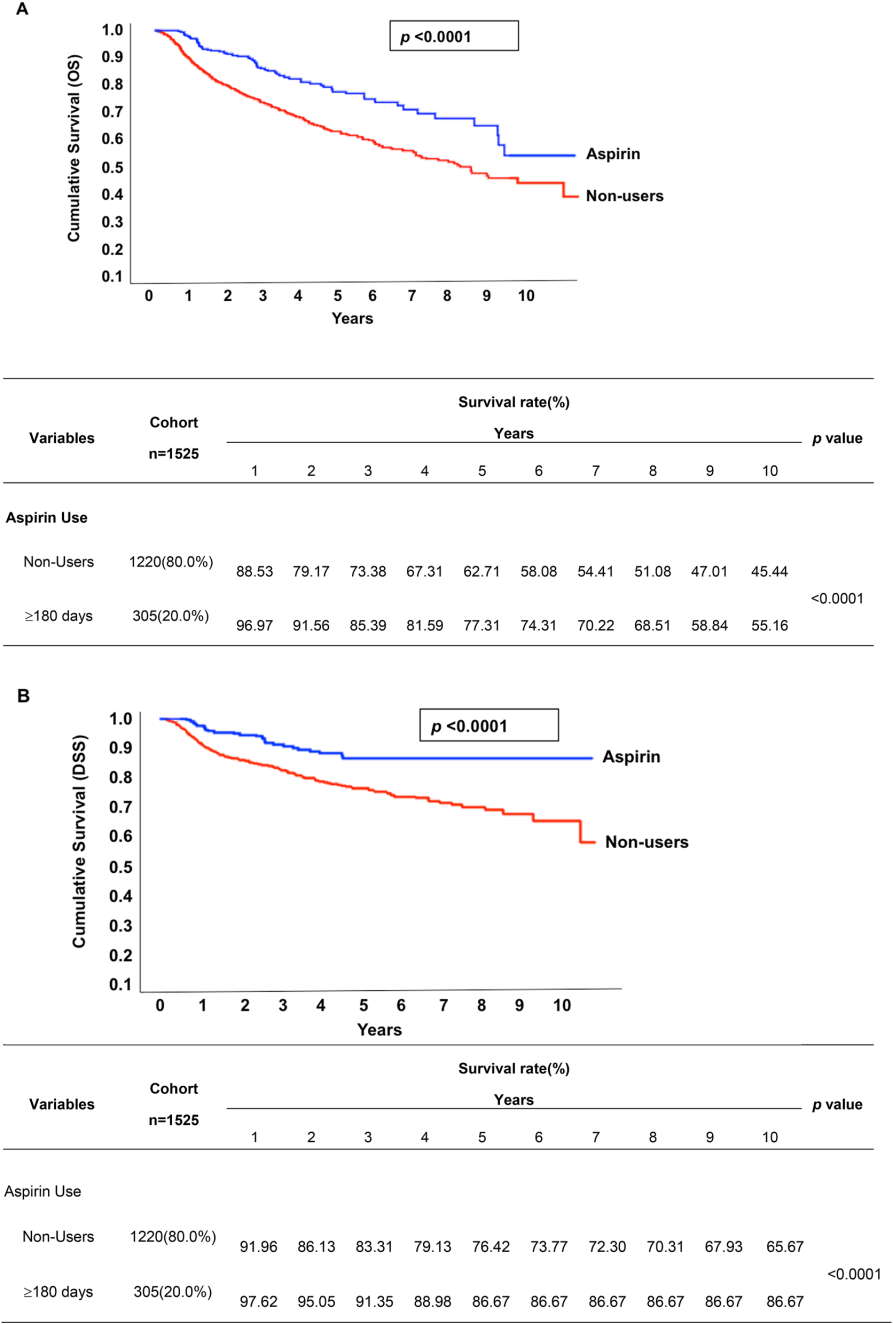
Kaplan–Meier survival curve of OS and DSS rates between aspirin users (≥ 180 days) and non-users. (**A**) The median OS rate of non-users was around 8 years and the estimated 10-year OS rate was 45.44%. Although median survival was not reached for aspirin users (≥ 180 days), estimated 10-year OS rate was 55.16%. (**B**) The estimated 10-year DSS rate of non-users was 65.67%, whereas aspirin users (≥ 180 days) was 86.67%.

There were 62 (20.33%) aspirin-using OSCC patients that died during the study period, while 404 (33.11%) deaths were of non-users (Table 1). Figure 2A,B show the Kaplan–Meier curves for overall survival (OS) and disease-specific survival (DSS) at 10 years in OSCC patients with and without 180-day aspirin use. Results of these analyses indicated that aspirin use is associated with improved OS and DSS within a 10-year follow-up period. Figure S1 shows no significant difference in DSS among the patients who took aspirin for fewer than 90 days or within 90–179 days, while OSCC patients who took aspirin consistently had better survival rates compared to non-users. We also evaluated the by comparing the cancer-specific (dead due to OSCC) and other-cause mortality (other causes of death) between aspirin users and non-users across different stages of cancer classified according to the pathological AJCC or TN staging system (Table 4). According to these analyses, the survival benefit conferred by aspirin use was observed in various stages of cancer, including pathological AJCC stages III & IV (*p* < 0.0001), T1, 2 & 3 (*p* = 0.0051), T4 (*p* = 0.0124) and N1, 2 & 3 (*p* = 0.0001) (Table 4).

**Table 4:** Comparing mortality rates between aspirin users (≥ 180 days) and non-users in OSCC patients (n = 1525) according to *different types of staging* systems.

Cancer staging system	Causes of death	N(%)	Low-dose aspirin		*p* value	
			Non-users n = 1220	Aspirin users ≥ 180 days n = 305		
**Pathological stages of cancer (AJCC)**^**a**^						
I & II	Alive	711(79.71%)	560(78.76%)	151(83.43%)	0.3283^e^	< 0.0001^k^
	Death due to OSCC	75(8.41%)	64(9.00%)	11(6.08%)		
	Other causes of death	106(11.88%)	87(12.24%)	19(10.50%)		
III & IV^b^	Alive	348(54.98%)	256(50.29%)	92(74.19%)	< 0.0001^f^	
	Death due to OSCC	189(29.86%)	171(33.60%)	18(14.52%)		
	Other causes of death	96(15.17%)	82(16.11%)	14(11.29%)		
**Pathological T staging**^c^						
T1, 2 & 3	Alive	905(74.24%)	696(72.27%)	209(81.64%)	0.0051^g^	< 0.0001^l^
	Death due to OSCC	161(13.21%)	141(14.64%)	20(7.81%)		
	Other causes of death	153(12.55%)	126(13.08%)	27(10.55%)		
T4	Alive	154(50.33%)	120(46.69%)	34(69.39%)	0.0124^h^	
	Death due to OSCC	103(33.66%)	94(36.58%)	9(18.37%)		
	Other causes of death	49(16.01%)	43(16.73%)	6(12.24%)		
**Pathological N staging**^**d**^						
N0	Alive	873(76.58%)	689(75.71%)	184(80.00%)	0.2179^i^	< 0.0001^m^
	Death due to OSCC	125(10.96%)	107(11.76%)	18(7.83%)		
	Other causes of death	142(12.46%)	114(12.53%)	28(12.17%)		
N1, 2 & 3	Alive	186(48.31%)	127(40.97%)	59(78.67%)	0.0001^j^	
	Death due to OSCC	139(36.10%)	128(41.29%)	11(14.67%)		
	Other causes of death	60(15.58%)	55(17.74%)	5(6.67%)		

^a^Pathological AJCC Cancer Staging 7th Edition.

^b^Stages IVa and IVb only.

^c^Pathological T Staging 7th Edition.

^d^Pathological N Staging 7th Edition.

^e–j^Aspirin non-users versus users.

^k^Stages I & II versus III & IV.

^l^T1, 2 & 3 versus T4.

^m^N0 versus N1, 2 & 3.

## Discussion

Some studies suggest that aspirin use has beneficial effects on head and neck squamous cell carcinoma (HNSCC)^16–19^, while other studies do not show this^20,21^. HNSCC is a collective term for carcinoma arising within the anatomic region of the head and neck, including the oral cavity, oropharynx, hypopharynx, nasopharynx and larynx. Although these tumors are all classified as squamous cell carcinomas, tumors that arise from the different anatomic regions of the head and neck differ in their epidemiology, tumor progression and therapeutic approach^1,12,21–24^. Thus, we paid particular attention to recruiting patients whose squamous cell carcinomas arose within the oral cavity, including the tongue, buccal area, etc. (Fig. S2).

Most, but not all, reports show an increased incidence of OSCC in men across different populations and geographic areas^12,25–28^*.* For example, OSCC occurred 1.45 times more frequently in men than in women in Japan^25^. Significant and consistent results in terms of OSCC affecting more men than women were found in countries such as Yemen, Taiwan and Pakistan^26–28^. One of the causes for the gender disparity in OSCC incidence is related to lifestyle behaviors, such as smoking and chewing betel nuts^29,30^. The prevalence rates of habits such as smoking, betel nut chewing and alcohol drinking habits are higher in men than in women^29,30^*.* However, a study from Taiwan had suggested that there is no difference in survival between men and women after being diagnosed with OSCC^29^. In our study, we noted that there is an 18-fold increase in the prevalence of OSCC in men relative to women, which is in agreement with the results showing that OSCC affects men more frequently than women^29,30^. Gender was considered one of the potential confounding variables in our study; we found that aspirin use still confers a survival benefit on patients with OSCC after controlling for this potential confounding variable.

A meta-analysis demonstrates that aspirin use confers a survival benefit in a dose-dependent manner, with a higher dosage of aspirin producing a greater reduction in the risk of colorectal cancer^31^. In addition, aspirin use has also shown benefits in reducing cancer in a duration-dependent manner across various cancers^17,32–34^. In our study, low-dose aspirin (100 mg/day) was prescribed and taken by patients after diagnosis of OSCC. We were able to access and trace the records for the duration of aspirin treatment of these OSCC patients by using the database from Chang Gung Memorial Hospital within a 10-year follow-up period. We classified the duration of aspirin use into three groups and examined whether the survival benefit of aspirin use appeared in a duration-dependent manner. The DSS rate of patients with OSCC who received aspirin for at least 180 days was significantly higher than that of non-users. In contrast, aspirin use had no significant effect on DSS in patients who only received aspirin for either less than 90 days or 90–179 days compared with non-users. Our results extend previous findings^16,32^ suggesting that over a 10-year follow-up period, aspirin use at a low dosage (100 mg) per day for at least 180 days has a survival benefit in patients with OSCC.

With the goal of providing more insight into whether aspirin has the potential to improve survival in OSCC patients, we included analyses of the associations between aspirin use and mortality rates in different stages of cancer according to the pathological AJCC or TN classification of malignant tumors. Patients who used aspirin for at least 180 days after diagnosis of OSCC showed significant improvement in 10-year OS and DSS compared with non-users, with a more marked benefit in late-stage OSCC (stages III & IV) (Table 4). However, the OS of aspirin users (55.16%) tended to be lower than DSS (80.67%) after 10 years (Fig. 2A,B). Inflammation is recognized as a driving force for the progression of OSCC and includes tissue invasion and metastasis, especially in late-stage OSCC^13,14^. Thus, the OS of aspirin users tending to be lower than the DSS of aspirin users after 10 years might imply that the treatment effect of aspirin is better at reducing OSCC-related death through regulating anti-inflammatory responses in OSCC patients.

Patients with T (T1, 2 & 3 and T4) and N (N1, 2 & 3) categories who took aspirin after diagnosis of OSCC were associated with increased survival rates (Table 4), while no significant survival differences were observed between aspirin users and non-users with OSCC who were classified as having an early stage of cancer (pathological AJCC stages I & II) and N0 (Table 4). Thus, our results suggest that OSCC patients who had advanced tumors, tumors spread to more lymph nodes, or regional metastases were more likely to benefit from aspirin use. Again, the results that we described above may be attributed to the anti-inflammatory effects of aspirin^14^. To date, it has been suggested that aspirin plays roles in anti*-*inflammatory effects through COX- and non-COX-dependent pathways^14,35^. COX is an enzyme that is responsible for the synthesis of prostaglandins from arachidonic acid^14,35,36^. There are two isoforms of this enzyme: COX-1 and COX-2^14,35,36^. It has been demonstrated that inhibition of COX-1 and/or COX-2 leads to decreased pro-inflammatory responses and/or reduced platelet aggregation^35,36^. Given that inflammation and platelet activation play important roles in cancer invasiveness and metastasis, patients with OSCC who have higher expression (mRNA and/or protein) levels of COX-1 and/or COX-2 might be very likely to benefit from aspirin use^13,14,35,36^.

It is not surprising that aspirin use has beneficial effects for patients who have the aforementioned tumor characteristics. The link between aspirin and cancer metastasis has been widely explored across various cancers^37–39^. Recently, a study demonstrated that aspirin prevents metastasis by disrupting the activation of endothelial cells, vasoconstriction, and the recruitment of monocytes to tumor sites through inhibition of the COX-1/TXA2 pathway^40^. The importance of this study along with the others is underscored by the fact that there is a phase III clinical trial, ADD-ASPIRIN, launching now that aims to evaluate the efficacy of aspirin in preventing metastases after patients have been diagnosed with breast, colorectal, gastroesophageal or prostate cancers. Hence, there is a clear possibility that aspirin has great potential to be an effective adjuvant cancer therapy for OSCC patients as well^41^.

## Conclusions, limitation and perspectives

Our study is an observational retrospective cohort study in which we looked at historical data for a group of OSCC patients with or without aspirin use. A limitation of such a retrospective cohort study is that the OSCC patients who were identified as aspirin users in this study were mostly those who showed signs and symptoms of cardiovascular diseases. For example, the OSCC patients who had risks for heart attack or stroke would be prescribed aspirin as part of the overall cancer treatment plan at the time they were diagnosed with OSCC. On the other hand, OSCC patients who had very little to no risk for heart attack or stroke would be less likely to take aspirin during their cancer treatment. For this reason, it was expected (as we noted) that our baseline patient characteristics would reflect this clinical setting, with prevalence of comorbidities being higher in aspirin users compared to non-users. With this limitation in mind, comorbidities were additionally adjusted in univariate and multivariate Cox regression analyses for controlling confounding effect. When comparing the Cox regression analyses of models with and without comorbidities as variables, aspirin use, pathological stages of cancer and treatment methods were still associated with OSCC survival even after adjusting for comorbidities. These results suggest that it is less likely that comorbidities would affect the outcome of our study.

Similarly, as a result of the inherent constraints of a retrospective cohort study with historical data, we could not assess the association of aspirin use with bleeding events in OSCC patients due to the lack of data on diagnoses of upper and lower gastrointestinal bleeding. Bleeding from a life-threatening condition such as a hemorrhagic stroke needs immediate emergency care^42,43^, meaning that we would receive this record in our database. However, as we looked at our study’s records of OSCC patients with a history of aspirin use, we did not identify any cases of hemorrhagic stroke that linked to aspirin use in OSCC patients. Numerous studies suggest that the benefit of aspirin use in some cancers is both dose- and duration-dependent^17,31–34^. We were able to classify the association of survival benefit with duration of aspirin use but unable to examine if there would be a greater survival benefit with an even shorter duration and higher dose of aspirin use. Since the use or longer duration of low-dose aspirin is still associated with a risk of bleeding^44^, the generalizability of our study might be perfected if we were able to identify whether the survival benefits of aspirin outweigh its potential risks in OSCC patients. This would require jointly examining the survival benefit of aspirin use in OSCC patients based on risk of bleeding, dose, and duration.

The results of this study demonstrate that during a 10-year follow-up period, aspirin use at a low dosage (100 mg) per day for at least 180 days confers a survival benefit on patients with OSCC. Given the poor prognosis of this disease, a treatment like aspirin that could not only improve the OSCC survival rate but is also inexpensive would be highly appreciated.

## Supplementary Information

Supplementary Information.
