# Supplementary material for: Low-dose aspirin confers a survival benefit in patients with pathological advanced-stage oral squamous cell carcinoma
Source: Sci Rep. 2021 Aug 25;11:17161. doi: 10.1038/s41598-021-96614-y (PMC8387371; doi:10.1038/s41598-021-96614-y)

**Low-dose aspirin confers a survival benefit in patients with pathological advanced-stage oral squamous cell carcinoma**

Sheng-Dean Luo, MD^1,2^, Shao-Chun Wu, MD^2,3^, Wei-Chih Chen, MD^1^, Ching-Nung Wu, MD^1^, Tai-Jan Chiu, MD^2,4^, Yao-Hsu Yang, MD ^5,6,7^, Shau-Hsuan Li, MD^4^, Fu-Min Fang, MD^8^, Tai-Lin Huang, MD^4^, Chang-Chun Hsiao, Ph.D.^2,9*^, Chang-Han Chen, Ph.D.^10,11,12,*^

^1^ Department of Otolaryngology, Kaohsiung Chang Gung Memorial Hospital and Chang Gung University College of Medicine, Kaohsiung 833, Taiwan.

^2^ Graduate Institute of Clinical Medical Sciences, College of Medicine, Chang Gung University, Taoyuan 333, Taiwan.

^3^ Department of Anesthesiology, Kaohsiung Chang Gung Memorial Hospital and Chang Gung University College of Medicine, Kaohsiung 833, Taiwan.

^4^ Department of Hematology-Oncology, Kaohsiung Chang Gung Memorial Hospital and Chang Gung University College of Medicine, Kaohsiung 833, Taiwan.

^5^ Department of Traditional Chinese Medicine, Chang Gung Memorial Hospital, Chiayi, Taiwan.

^6^ Health Information and Epidemiology Laboratory of Chang Gung Memorial Hospital, Chiayi, Taiwan.

^7^ School of Traditional Chinese Medicine, College of Medicine, Chang Gung University, Taoyuan, Taiwan.

^8^ Department of radiation Oncology, Kaohsiung Chang Gung Memorial hospital and Chang Gung University College of Medicine, Kaohsiung, Taiwan.

^9^ Division of Pulmonary and Critical Care Medicine, Kaohsiung Chang Gung Memorial Hospital and Chang Gung University College of Medicine, Kaohsiung 83301, Taiwan

^10^ Department of Applied Chemistry, and Graduate Institute of Biomedicine and Biomedical Technology, National Chi Nan University, Nantou 54561, Taiwan.

^11^ Institute of Medicine, Chung Shan Medical University, Taichung, Taiwan.

^12^ Department of Medical Research, Chung Shan Medical University Hospital, Taichung, Taiwan.

^*^ Corresponding Authors:

Dr. Chang-Chun Hsiao

Graduate Institute of Clinical Medical Sciences, College of Medicine, Chang Gung University, Taoyuan, Taiwan.

Address: No. 259, Wenhua 1st Rd., Guishan District, Taoyuan City, 333, Taiwan

E-mail: cchsiao@mail.cgu.edu.tw

Dr. Chang-Han Chen

Department of Medical Research, Chung Shan Medical University Hospital, Taichung, Taiwan.

Address: No.110, Sec.1, Jianguo N. Rd., Taichung City 40201, Taiwan.

E-mail: chench7@gmail.com; [changhan155@hotmail.com](mailto:changhan155@hotmail.com)

**Supplemental Results**

**Table S1. Criterion for OSCC Patients who had Unhealthy Habits and Comorbidities.**

| **Variables** | **Criterion** |
| --- | --- |
| **Lifestyle Risk Factors** |  |
| Smoking | Patients who had smoked more than 100 cigarettes during their lifetime and smoked daily or some days. |
| Betel nut chewing | Patients who had chewed betel nuts more than 6 months and chewed betel nut daily or some days. |
| Alcohol consumption | Patients who had drunk alcoholic beverages daily or on some days. |
| **Comorbidities** |  |
| Atrial fibrillation (flutter) | Based on the results of ECG, which recorded the heart rhythms and electrical activities of OSCC patients. |
| DM | Based on one or more of the results of A1C, fasting blood glucose, glucose tolerance or random blood sugar test. |
| Hypertension | Based on the results of a 24-hour blood pressure monitoring test. |
| Hyperlipidemia | Based on the results of a blood test for levels of cholesterol. |

|  | **Number of Subjects (A)** | |  | **Number of Missing Subjects (B)** | |  | **Total (A+B)** | |
| --- | --- | --- | --- | --- | --- | --- | --- | --- |
|  | **N** | **%** |  | **N** | **%** |  | **N** | **%** |
| **Survival Time (Month)** | 1525 | 100.0% |  | 0 | 0.0% |  | 1525 | 100.0% |

**Table S2. Survival time (follow-up duration) of OSCC Patients.**

**Descriptive Statistics**

|  | | |  | **Value** | **Standard Error** |
| --- | --- | --- | --- | --- | --- |
| **Survival Time (Month)** | **Mean**  **(95% Confidence Interval)** | 58.997  (57.208-60.785) | 0.9117 | | |
|  | **5% Modified Mean** | 58.099 |  | | |
|  | **Mean** | 56.867 |  | | |
|  | **Variation** | 1267.486 |  | | |
|  | **Standard Deviation** | 35.6018 |  | | |
|  | **Minimum** | 1.9 |  | | |
|  | **Maximum** | 133.8 |  | | |
|  | **Range** | 131.8 |  | | |
|  | **Interquartile Range (IQR)** | 58.6 |  | | |
|  | **Skewness** | 0.268 | 0.063 | | |
|  | **Kurtosis** | -0.956 | 0.125 | | |

Percentile

|  |  | **5** | **10** | **25** | **50** | **75** | **90** | **95** |
| --- | --- | --- | --- | --- | --- | --- | --- | --- |
| **Survival Time (Month)** | **Weighted Average** | 8.210 | 12.400 | 27.400 | 56.867 | 86.017 | 110.427 | 122.790 |
|  | **Tukey Hinges** |  |  | 27.400 | 56.867 | 85.967 |  |  |

**Table S3. Baseline Characteristics of OSCC Patients Before and After Propensity-Score Matching (PSM).**

| **Characteristics** | **Before Propensity Score Matching** | | | | |  | | **After Propensity Score Matching** | | | |
| --- | --- | --- | --- | --- | --- | --- | --- | --- | --- | --- | --- |
|  | Non-users    (Control)  n=6018 | | Aspirin Users  ≥180 days  (Intervention)  n=306 | | SMD |  | Non-users    (Control)  n=1220 | | | Aspirin Users  ≥180 days  (Intervention)  n=305 | SMD |
| **Propensity Score (IQR)** | 0.036(0.022-0.059) | | 0.064(0.041-0.102) | | 0.6874 |  | | | 0.063(0.041-0.103) | 0.063(0.041-0.102) | 0.0098 |
| **Gender** |  | |  | |  |  | | |  |  |  |
| Female (%) | 521(8.66%) | | 20(6.54%) | | 0.0801 |  | | 58(4.75%) | | 20(6.56%) | 0.0780 |
| Male (%) | 5497(91.34%) | | 286(93.46%) | | 0.0801 |  | 1162(95.25%) | | | 285(93.44%) | 0.0780 |
| **Age (IQR)** |  | |  | |  |  | | |  |  |  |
| Years | 52(45-59) | | 58(53-65) | | 0.6154 |  | 58(52-65) | | | 58(53-65) | 0.0072 |
| **Pathological Stages**  **of Cancer (AJCC)^a^** | | | | |  |  | |  | |  |  |
| I & II(%) | | 2882(47.89%) | | 182(59.48%) | 0.2338 |  | | 711(58.28%) | | 181(59.34%) | 0.0216 |
| III & IV^b^ (%) | | 3136(52.11%) | | 124(40.52%) | 0.2338 |  | | 509(41.72%) | | 124(40.66%) | 0.0216 |
| **Treatments** | |  | |  |  |  | |  | |  |  |
| Surgery | | 3224(53.57%) | | 177(57.84%) | 0.0860 |  | | 681(55.82%) | | 176(57.7%) | 0.0380 |
| Surgery & RT or CCRT | | 2794(46.43%) | | 129(42.16%) | 0.0860 |  | | 539(44.18%) | | 129(42.3%) | 0.0380 |

**Abbreviations:** SMD Standardized Mean Difference; RT radiotherapy; CCRT concurrent chemoradiotherapy.

^a^ Pathological AJCC Cancer Staging 7^th^ Edition;

^b^ Stages IVa and IVb only.

**Table S4. Univariate and multivariate Cox proportional hazards of prognostic**

**factors for OSCC survival, Model C: adjusted additional to all comorbidities and the covariates in Model A.**

| **Variables** | **Cohort**  **n=1525** |  | | **Hazard Ratio (95%CI)** | | |
| --- | --- | --- | --- | --- | --- | --- |
|  |  | Univariate | *p*-value | | Multivariate | *p*-value |
| **Gender** |  |  |  | |  |  |
| Female | 78(5.11%) | 1 | 0.0843 | | 1 | 0.3104 |
| Male | 1447(94.89%) | 1.94(0.91-4.11) |  |  | 1.48(0.69-3.17) |  |
| **Age** |  |  |  | |  |  |
| Years | 1525(100.00%) | 1.00(0.99-1.01) | 0.7096 | | 1.01(0.99-1.02) | 0.2814 |
| **Pathological Stages of Cancer (AJCC)^a^** |  |  |  | |  |  |
| I & II | 892(58.5%) | 1 | <0.0001 | | 1 | <0.0001 |
| III & IV^b^ | 633(41.5%) | 4.22(3.23-5.52) |  |  | 2.34(1.66-3.30) |  |
| **Treatments** |  |  |  | |  |  |
| Surgery | 857(56.2%) | 1 | <0.0001 | | 1 | <0.0001 |
| Surgery & RT or CCRT | 668(43.8%) | 4.31(3.27-5.68) |  |  | 2.60(1.83-3.70) |  |
| **Aspirin Use** |  |  |  | |  |  |
| No | 1220(80.0%) | 1 | <0.0001 | | 1 | 0.0191 |
| Yes | 305(20.0%) | 0.44(0.30-0.64) |  |  | 0.59(0.38-0.92) |  |
| **Comorbidities** |  |  |  | |  |  |
| Atrial fibrillation (flutter) |  |  |  | |  |  |
| No | 1483(97.25%) | 1 | 0.5779 | | 1 | 0.9376 |
| Yes | 42(2.75%) | 0.80(0.35-1.79) |  |  | 1.03(0.45-2.40) |  |
| DM |  |  | 0.6002 | |  | 0.0022 |
| No | 1171(76.79%) | 1 |  |  | 1 |  |
| Yes | 354(23.21%) | 0.93(0.69-1.24) |  | | 1.65(1.20-2.28) |  |
| Hypertension |  |  |  |  |  |  |
| No | 1082(70.95%) | 1 | 0.0084 | | 1 | 0.8841 |
| Yes | 443(29.05%) | 0.68(0.51-0.91) |  |  | 0.98(0.71-1.34) |  |
| Hyperlipidemia |  |  |  | |  |  |
| No | 1221(80.07%) | 1 | <0.0001 | | 1 | <0.0001 |
| Yes | 304(19.93%) | 0.32(0.21-0.48) |  |  | 0.33(0.20-0.53) |  |

**Abbreviations:** 95% CI 95% confidence interval; RT radiotherapy; CCRT concurrent chemoradiotherapy; DM diabetes mellitus.

^a^ Pathological AJCC Cancer Staging 7^th^ Edition;

^b^ Stages IVa and IVb only.


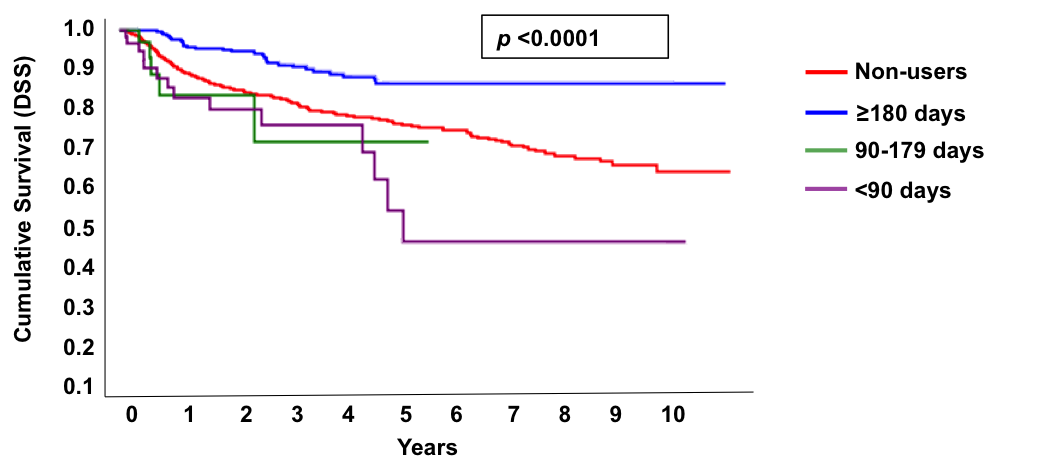


| **Variables** | | **Cohort**  **n=2020** | **Survival rate(%)**  **Years** | | | | | | | | | | ***p* value** |
| --- | --- | --- | --- | --- | --- | --- | --- | --- | --- | --- | --- | --- | --- |
|  |  |  | 1 | 2 | 3 | 4 | 5 | 6 | 7 | 8 | 9 | 10 |  |
| **Aspirin Use** |  | |  |  |  |  |  |  |  |  |  |  |  |
| Non-Users | 1616(80.0%) | | 90.91 | 85.28 | 82.55 | 78.59 | 76.30 | 74.85 | 71.36 | 68.39 | 65.96 | 64.27 | <0.0001 |
| ≥180 days | 304(15.05%) | | 97.61 | 95.04 | 91.32 | 88.93 | 86.61 | 86.61 | 86.61 | 86.61 | 86.61 | 86.61 |  |
| 90-179 days | 36(1.78%) | | 83.77 | 83.77 | 71.80 | 71.80 | 71.80 | 71.80 |  |  |  |  |  |
| <90 days | 64(3.17%) | | 82.90 | 79.90 | 76.26 | 76.26 | 54.60 | 46.80 | 46.80 | 46.80 | 46.80 | 46.80 |  |

**Figure S1. Kaplan-Meier survival curve of disease-specific survival (DSS) among non-users, ≥180-day, 90-179-day and <90-day aspirin users.** A total of 2,020 patients diagnosed with OSCC were recruited for this study after 1:4 PSM. The estimated 5- and 10-year DSS rates of non-users were 76.307% and 64.27%, respectively. The estimated 5- and 10-year DSS rates of ≥180-day aspirin users were both 86.61%. The estimated 5-year DSS rate of 90-179-day aspirin users was 71.8%. The estimated 5- and 10-year DSS rates of <90-day aspirin-users were 54.60% and 46.80%, respectively. *P-*value was 0.0001 for the overall comparison among the groups using the log rank test.

Figure S2. Pie chart showing locations (%) of OSCC found in patients.


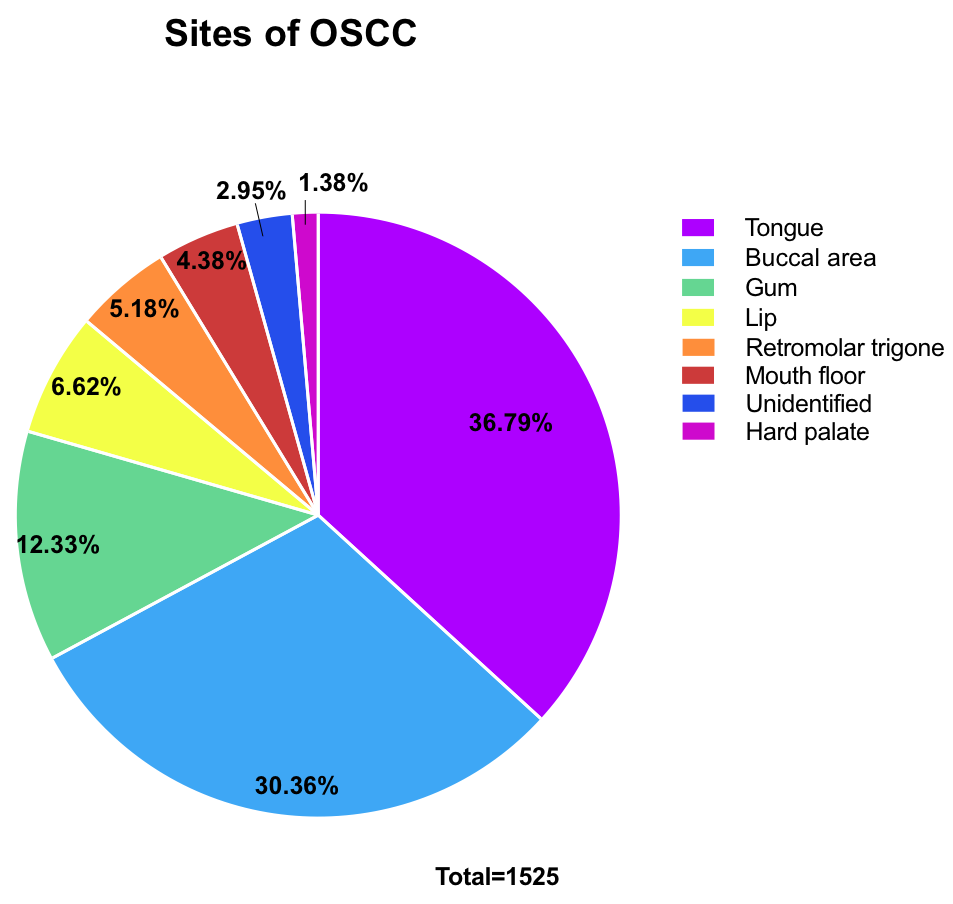

Supplement: Supplementary file 1 — Supplementary Information. [file 41598_2021_96614_MOESM1_ESM.doc]
